# Factors associated with relevant knowledge of intestinal schistosomiasis and intention to participate in treatment campaigns: a cross sectional survey among school children at Ijinga Island on Lake Victoria, North-Western Tanzania

**DOI:** 10.1186/s12889-019-8091-4

**Published:** 2019-12-30

**Authors:** Sandra Parisi, Humphrey D. Mazigo, Saskia Kreibich, Karl Puchner, Christa Kasang, Andreas Mueller

**Affiliations:** 1 DAHW - German Leprosy and Tuberculosis Relief Association, Raiffeisenstrasse 3, 97080 Würzburg, Germany; 2 School of Medicine, Department of Medical Parasitology, Catholic University of Health and Allied Sciences, P.O. Box 1464, Mwanza, Tanzania; 3 Medical Mission Institute, Salvatorstrasse 7, 97067 Würzburg, Germany; 4 Department of Tropical Medicine, Medical Mission Hospital, Salvatorstrasse 7, 97074 Würzburg, Germany

**Keywords:** Schistosomiasis, Relevant knowledge, Intention, Mass drug administration, Protection motivation theory

## Abstract

**Background:**

Annual Mass Drug Administration (MDA) using praziquantel targeting primary school children is the main control strategy against schistosomiasis in Tanzania. However, there are concerns about decreasing participation in mass drug administration among primary school children for unknown reasons. Therefore, the aim of this study was to identify factors related to relevant knowledge about schistosomiasis and the intention to participate in mass drug administration among primary school children in order to give recommendations for future projects.

**Methods:**

A cross sectional, extended knowledge, attitudes and practices (KAP) survey was conducted among 356 primary school children aged 5–17 years in February–March 2016 using a pre-tested questionnaire. This survey was part of a baseline assessment for an integrated proof of concept study aiming towards schistosomiasis elimination on Ijinga Island. Outcomes of interest in logistic regression analysis were relevant knowledge and high intention to participate in treatment campaigns. Explanatory variables were sociodemographic information sources and elements aligned to Protection Motivation Theory (PMT).

**Results:**

Only 17% of the children had relevant intestinal schistosomiasis related knowledge and very few of them knew any of the *S. mansoni* manifestations and complications. Factors associated with relevant schistosomiasis knowledge were previous diagnosis of schistosomiasis (aOR = 2.43, 95%CI: 1.1–5.6), having heard about schistosomiasis at school (aOR = 9.94, 95%CI: 5.0–19.7) and being enrolled in 6th or 7th grade (aOR = 3.94, 95%CI: 1.3–11.8). Only 40% of the children demonstrated high intention to participate in treatment campaigns. Factors associated with high intention to participate in MDA were previous diagnosis (aOR = 2.23, 95%CI: 1.1–4.7), perceived general risk of disease transmission by lake water (aOR = 1.79, 95%CI: 1.0–3.1), perceived own vulnerability of getting infected (aOR = 5.10, 95%CI: 2.1–12.6), perceived danger of the disease (aOR = 2.47, 95%CI: 1.3–4.8) and the perceived effectiveness of medicaments to cure the disease (aOR = 2.86, 95%CI: 1.4–5.7).

**Conclusions:**

The minority of the school children had high level of theoretical knowledge about schistosomiasis and a small proportion of the children demonstrated high intention to participate in mass drug administration. In general, practical knowledge on preventive measures such as taking anti-schistosomiasis drug during MDA need to be impacted in school children to increase their participation in the control program.

## Background

Schistosomiasis is one of the neglected tropical diseases, which is highly prevalent in the tropical and sub-tropical areas of the African continent [1, 2]. Worldwide, an estimated 779 million people live in areas characterized with high transmission of schistosomiasis and 290 million people are estimated to be infected with the disease, 93% of them are found in Sub-Saharan Africa (SSA) [1, 2]. Approximately, 120 million people have schistosomiasis related manifestations and over 2.8 million years lived with disabilities are attributed to schistosomiasis [3].

In SSA, schistosomiasis is caused by two major blood trematodes, *Schistosoma mansoni* causing intestinal schistosomiasis and *Schistosoma haematobium* causing urogenital schistosomiasis [1]. In central Africa, *S. intercallatum* is an additional causative agent of schistosomiasis. *Schistosoma mansoni* causes gastrointestinal related symptoms and related chronic disease stages are associated with hepatomegaly, periportal fibrosis possibly leading to portal hypertension, oesophageal varices and hematemesis (blood vomiting) [4]. It is estimated that in the African continent, 8.5 million cases of chronic hepatosplenic schistosomiasis are related to *S. mansoni* infection [4]. The primary clinical manifestations of urogenital schistosomiasis are haematuria, dysuria, urinary bladder pathology and hydronephrosis [4]. An estimated 10 million cases of hydronephrosis in SSA are attributed to *S.haematobium* infection [4]. Besides the above mentioned morbidities, chronic schistosomiasis in school children can additionally cause anaemia, stunted growth and other signs of under-nutrition resulting into impaired cognitive development, which affects their school performance [5]. In SSA, because of high water contacts behaviors and frequent re-infections, school children carry the highest burden of disease, but depending on the geographical location, adult individuals can also harbor high intensities of infection [1, 6, 7]. Moreover, during the past two decades, data have emerged showing a high prevalence and intensity of infection among pre-school aged children (PSAC) [8, 9].

After Nigeria, Tanzania is the country with the second highest burden of schistosomiasis in SSA [10]. In 2012, approximately 52% (23 million people) of the Tanzanian population were estimated to be infected with schistosomiasis [10]. Both, *S.mansoni* and *S.haematobium,* are endemic in the country to a varying extent depending on the geographical region [6]. The area along Lake Victoria - the northwestern region - is highly affected, with areas located along the lake shore and the islands being endemic for *S.mansoni* [6]. Areas located more in the inlands are endemic for *S.haematobium* [6]. The main control approach against schistosomiasis in Tanzania focusses on annual mass drug administration with praziquantel [11]. The only focus of the MDA program are school children due to low associated costs and the fact that school-aged children carry the heaviest burden of infection compared to other members of the community [11]. Since 2006, multiple rounds of MDA campaigns have been conducted in Tanzania [6], but high transmission rates of schistosomiasis continue to persist throughout the country [6]. This observation indicate that standalone MDA will not interrupt the infection cycle and this highlights the need for integrated control approaches targeting at sustained situational improvements [12]. Such integrated control measures should involve public health education, improved socio-economic living conditions in the communities, engagement of local communities in preventive action and treatment, improvement of water, sanitation and hygiene (WASH), as well as a general integration of preventive schistosomiasis services in local health systems [11].

Another important challenge of MDA campaign is acceptance among the targeted population towards the treatment. Previous studies among the adult population have indicated poor uptake and unwillingness to take preventive chemotherapy [13–15]. In Uganda, self-reported uptake of preventive treatment against schistosomiasis and soil-transmitted helminths in school children was only 28.2% [16] and in central Tanzania was only 43.6% [17]. A number of factors have been described to account for low and high uptake of preventive chemotherapy [18]. Low uptake of preventive chemotherapy among school children was mainly associated with: inadequate preparation of teachers to offer MDA to school children; children’s fear of side effects; inadequate communication regarding the MDA rationale; insufficient knowledge about MDA and schistosomiasis disease; deliberate absenteeism from school during the treatment; and lack of teachers support [16, 19, 20]. On the other hand, a good level of received information and knowledge of MDA campaigns and schistosomiasis, supportive teachers as well as the provision of food prior to drug distribution was shown to increase the uptake rates of preventive chemotherapy among school children [16, 19, 20].

The integration of public health topics such as schistosomiasis and its related preventive measures into the educational curriculum of school children improves relevant knowledge about the disease and promotes preventive behavior such as improved participation in treatment campaigns [5]. The primary school curriculum of Tanzania covers some aspects of common diseases (including schistosomiasis and their preventive measures) for sixth and seventh grade, but the question remains if this strategy is sufficient in high endemic settings, where the risk for heavy infection starts already at pre-school age and continues throughout adulthood [21, 22]. It is therefore relevant to determine, whether school children have adequate knowledge to seek secondary prevention once they are no longer targeted by national MDA campaigns.

In this context, the present study aimed at identifying factors associated with relevant schistosomiasis knowledge and the intention to participate in treatment campaigns among school children on Ijinga Island, northwestern Tanzania. Although very important, studies from China showed that education campaigns targeting knowledge alone do not consequently lead to behavior change [23, 24]. There seems to be a gap between knowledge and the resulting practices concerning preventive behavior. Due to the recognized limitations of a KAP assessment this study included elements of Protection Motivation Theory (PMT) [25], aligning some questions to a behavior change theory-based framework to explore its potential relevance within the Tanzanian context for future studies. According to PMT the intention to adapt protective behavior depends on an appraisal of threat caused by a disease and one’s individual ability to cope with this threat. We therefore included potential factors such as perceived vulnerability, severity and response efficacy in the analysis to give a more comprehensive picture on the outcome of interest.

## Methods

### Study area and population

The study was conducted on Ijinga Island located at the southern shoreline of Lake Victoria, Magu district, Mwanza region, northwestern Tanzania. According to the population census obtained at the village in 2016, the village has a total of 400 households inhabited by 2520 residents and among them, 600 are primary school children. The island has one dispensary and one primary school. The main economic activities of the inhabitants are subsistence farming (rice, maize and cassava), livestock keeping and fishing. The island has five sub-villages, namely Kashishi, Mwamalangale, Ngambaji, Ilago and Igadi. The main source of water for domestic and recreational use and irrigation is the Lake Victoria. The ecology of the island supports the transmission of intestinal schistosomiasis. Data from the Schistosomiasis Consortium for Operational Research and Evaluation (SCORE) project from the years 2013 and 2014 showed that 80% of the school-aged children were infected with intestinal schistosomiasis (using the Kato Katz technique), and an own assessment in 2016 using point-of-care Circulating Cathodic Antigen test revealed that 97% of the tested children had *S. mansoni* infection (unpublished data). School children receive annual mass drug administration against schistosomiasis using praziquantel with minimal health education provided by school teachers who participate in treating children. For the past 5 years (2010–2015), the school has been receiving one round of MDA annually. Data from the District Neglected Tropical Diseases Control coordinator indicate that the treatment coverage against schistosomiasis at Magu district was only 70% (district NTD coordinator data unpublished), which was below the WHO recommendation of 75%. Specifically, the uptake of PZQ at Ijinga Primary school for the same period has been below 50% (district NTD coordinator data).

### Study design and inclusion criteria

This study was designed as a cross-sectional extended KAP-survey, which was conducted in February and March 2016 and involved primary school children attending Ijinga primary school. The study used a pre-tested questionnaire which was validated in a different community. Children were eligible to participate in the study if: (i) they were attending the selected school; (ii) they were aged between 5 and 17 years; (iii) they provided signed informed consent from parents/ guardians; (iv) they were able to express themselves; and (v) were present at the day of interview.

### Sample size and sampling procedures

The Kish Leslie Formula was used to determine the minimum sample size [26]. General awareness on schistosomiasis has been noted to range between 45 and 50% in previous studies in communities living around the great lake areas [27, 28]. Considering the prevalence of relevant schistosomiasis knowledge of 45% [27], at 95% confidence interval and margin errors (tolerable error) of 5%, a minimum sample of 346 school children was required. However, 356 school children met the inclusion criteria and were enrolled in the study. Using the class attendance register, children were chosen using a systematic sampling procedure as described elsewhere [16].

### Data collection

#### Interview using the questionnaire

Trained research assistants individually administered the questionnaire (Additional file 1) to school children. The questionnaire had open and closed ended questions and collected information on knowledge about schistosomiasis, signs and symptoms and preventive measures. Other questions were on perceived risk for schistosomiasis, perceived benefits of treatment, treatment seeking behaviors and praziquantel side effects. Prior to data collection, the questionnaire was pre-tested at Igombe and Kabangaja primary schools located in Ilemela district. After pre-testing, corrections were done and the corrected version of the questionnaire was used for data collection. Some questions were aligned to elements of Protection Motivation Theory [25], in order to further explore the knowledge-attitude- behavior continuum.

### Study outcomes and data analysis

Outcomes of interest were relevant knowledge and intention to participate in treatment campaigns. In order to measure relevant knowledge, a score was created based on the number of correct answers given to five questions related to transmission and clinical manifestation of schistosomiasis. Answers were cited spontaneously to identify true awareness, without reading out options (multiple answers possible). In order to define a minimum level of relevant theoretical knowledge, a binary variable was created taking a cut off value of five points, indicating an average of at least one correct answer per question/ topic of interest (types of schistosomiasis, clinical manifestation, area of transmission, ways of getting infected and passing the disease to others). In order to measure intention of participating in treatment campaigns, a score was created using the answers to three questions (*would you participate in test and treat campaigns, would you participate in MDA, would you advise your friend to participate in MDA*). These questions were not asked consecutive, but in different phases of the interview. Many children gave inconsistent answers to the three questions (potentially influenced by lack of knowledge, previous questions and social desirability bias). Therefore, to assure true motivation, a binary outcome variable for high intention to participate in treatment campaigns was created for those with consistent positive answers in all three questions. Explanatory variables of interest were: (i) sociodemographic variables such as sex, age, class and migration status, (ii) previous schistosomiasis diagnosis or participation in MDA (iii) information source about schistosomiasis (school; village/family members; health care system; local media) and (iv) variables aligned to elements of protection motivation theory such as perceived severity, perceived vulnerability and perceived efficacy of treatment.

All collected information using the questionnaire were entered into a data entry form (Epidata) and subsequently transferred to an Excel sheet for cleaning purposes. To avoid systematic errors, all data were double entered. Statistical analysis, comprising of descriptive and regression analyses, was done by using STATA 13 (StataCorp, 2017, Stata statistical software: release 13. College Station, TX: StataCorp LP, Taxes, USA). Bivariate and multivariate analyses using logistic regression were performed in order to determine factors associated with relevant knowledge and high intention of participating in treatment campaigns. At bivariate level, all factors with *P*-value of < 0.2 were considered for multivariate analysis. A *p*-value of < 0.05 was considered statistically significant. Likelihood ratio tests were performed in order to determine the significance of factors and their interactions in the final model.

## Results

### Demographic characteristics of the study participants

Overall, a total of 356 school children aged 5–17 years were enrolled in the study. Of these children, 47 and 53% were female and male respectively (Table 1). The vast majority of the children (87%) were born on Ijinga Island and almost all of them (92%) reported to have regular lake water contact. The main reported reasons for lake water contact were bathing, swimming and playing (*n* = 216), fetching water (*n* = 148) and washing cloths and utensils (*n* = 112). Most children (83%) reported that drinking water was cleaned through filtration and/or boiling before use in their households. In general, 94% of the children reported that their households had toilets and that they were used by household members. The majority of the children (71%) believed that the use of toilets could prevent the transmission of disease.

**Table 1:** Socio demographic information of children attending Ijinga primary school, northwestern Tanzania

Variable	Frequency	Percent (%)
Sex		
Male	187	52.53
Female	169	47.47
Age Group		
5–9	83	23.31
10–13	182	51.12
14–17	91	25.56
Class enrolled at school		
1–3	162	45.63
4–5	111	31.27
6–7	82	23.10
Migration Status		
Born in Ijinga	307	86.97
Migrated to Ijinga	46	13.03

### Awareness of schistosomiasis

**Fig. 1:**
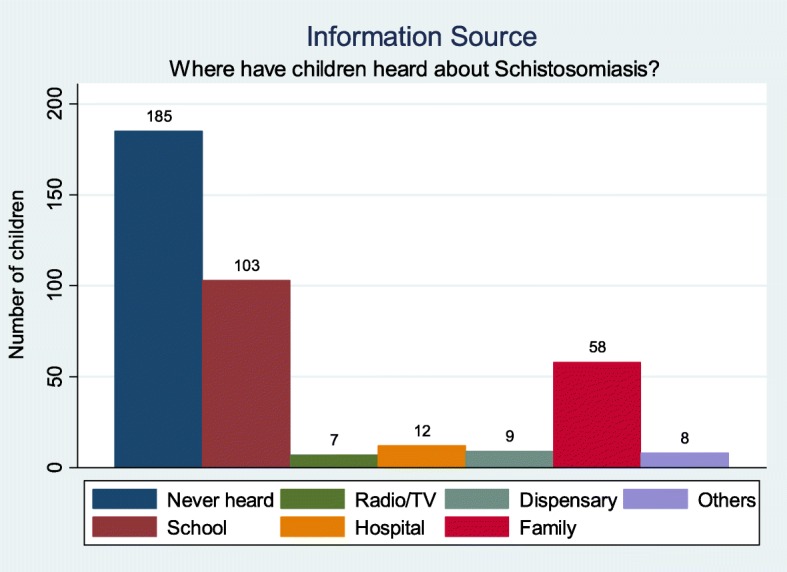
Source of information about schistosomiasis among school children at Ijinga Island, northwestern Tanzania

Even though Ijinga island is a highly endemic setting for schistosomiasis, more than half (52%) of the surveyed school children reported to have never heard about the infection/disease. For those children who have heard of schistosomiasis, the majority (61%, *n* = 103, *N* = 170) mentioned the school as source of information about schistosomiasis. The other sources of information about schistosomiasis are shown in Fig. 1.

### Knowledge about symptoms and clinical signs of schistosomiasis

**Fig. 2:**
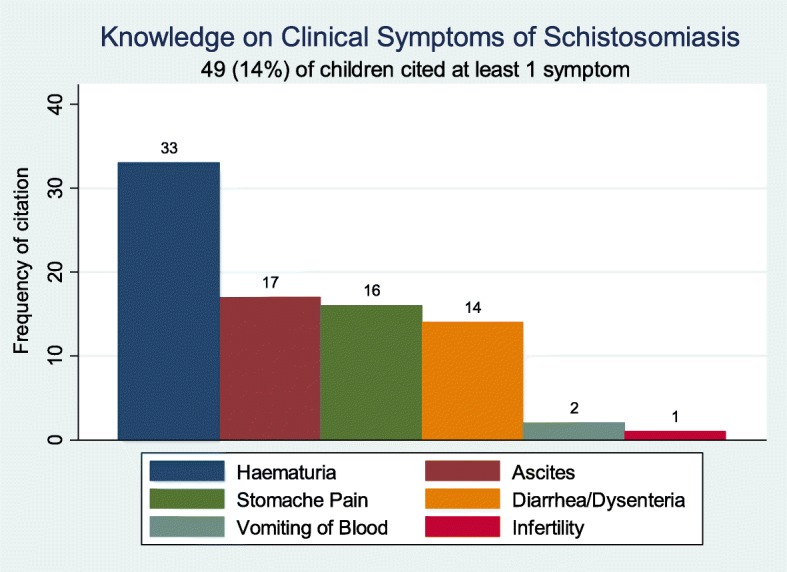
Most common schistosomiasis symptoms and signs mentioned by surveyed school children on Ijinga Island

With regard to knowledge about clinical symptoms, only 14% of the children were able to mention at least one correct clinical symptom associated with intestinal schistosomiasis (Fig. 2). In addition, only 10% of the children knew the two types of schistosomes which are endemic in Tanzania – *S. mansoni* and *S. haematobium* causing intestinal and urogenital infection/disease. The most commonly cited clinical symptom was hematuria, followed by more unspecific symptoms of *S. mansoni* (ascites, stomach pain, diarrhea/ dysentery, presented in Fig. 2).

When asked if they had previously experienced any schistosomiasis related symptoms, only few children mentioned hematuria (*n* = 5), stomach aches (*n* = 4) and stomach swelling (*n* = 2) being the common symptoms they have ever experienced. Moreover, 16% (*n* = 56) of the children indicated a previous diagnosis for schistosomiasis, with the majority being diagnosed at the hospital (*n* = 49). Seven out of the 56 children mentioned a diagnosis through medical researchers at the school environment. Most children with previous schistosomiasis diagnosis (*n* = 47, *N* = 56; 83.9%) reported to have received treatment.

### Knowledge about transmission mode and preventive measures of schistosomiasis

**Fig. 3:**
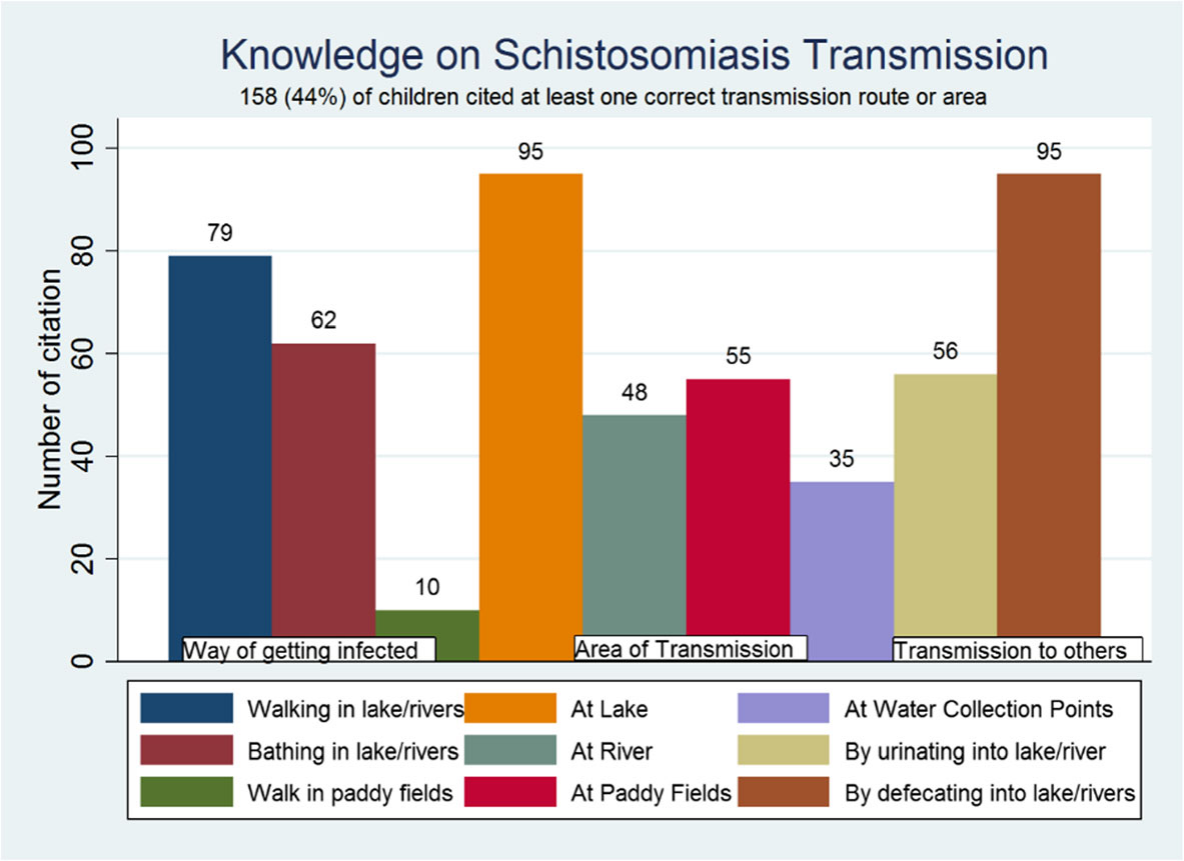
Knowledge on schistosomiasis transmission among by surveyed school children on Ijinga Island

In general, knowledge on the mode of transmission of schistosomiasis was relatively high, with 44% (*n* = 158) of the school children mentioning at least one correct transmission pathway (Fig. 3). However, also misconceptions regarding the modes of schistosomiasis transmission were prevalent among the study population, with children stating that transmission can occur in toilets (*n* = 36, 10%) and through the consumption of contaminated food (*n* = 15, 4%).

In relation to knowledge about preventive measures, 40% (*n* = 143) of the children cited at least one correct way of preventing schistosomiasis. The mentioned preventive measures were avoiding lake water contact (*n* = 102, 29%), taking drugs (*n* = 43, 12%) and using gum boots when working in water sources (*n* = 16, 4%) (Fig. 3). However misconceptions were also mentioned when asking about preventive and control measures, such as boiling and filtering drinking water before use (*n* = 26), eating uncontaminated food (*n* = 17) and cleaning the body (*n* = 8) or environment (*n* = 14).

### Level of relevant knowledge about schistosomiasis

**Fig. 4:**
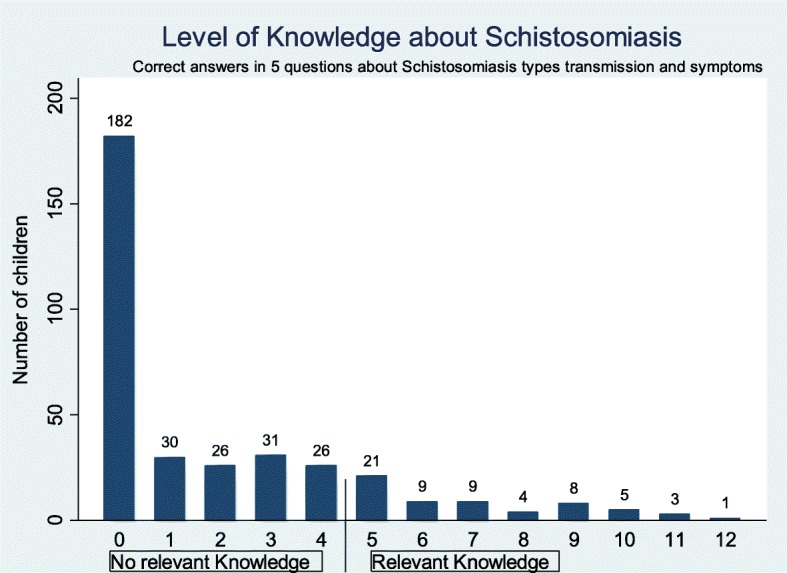
Level of relevant knowledge about schistosomiasis and its mode of transmission among primary school children on Ijinga Island

Based on our analysis, using a sum of correct answers (cited spontaneously), the mean knowledge score among the surveyed school children of Ijinga island was 1.97 ± 2.7 (range: 0–12 points), (Fig. 4**)**. Approximately half (51%) of the children did not give any correct answer. Based on our definition, only 17% (*n* = 60) of the children had relevant knowledge about schistosomiasis and its transmission (Fig. 4).

### Attitudes and practices towards schistosomiasis and the intention to participate in treatment campaigns

Of all the respondents, 41% (*n* = 143/356) believed that lake water contact was the source of any disease. When asked if they consider themselves at risk of contracting schistosomiasis, 12% (*n* = 41) considered themselves to be at high risk, 55% (*n* = 192) at low risk, and 33% (*n* = 115) at no risk.

The majority of children (*n* = 229, 65%) considered schistosomiasis to be a dangerous disease and about half of the children (*n* = 189, 53%) believed that schistosomiasis can lead to death. Overall, 67% (*n* = 238) of the children mentioned that the drug praziquantel can cure schistosomiasis and 33% (*n* = 116) believed in traditional medicine. A total of 26% (*n* = 90) of the children thought that the schistosomiasis medication has side effects and 23% (*n* = 80) even believed that those drugs could cause death.

Approximately one third of the interviewed children (*n* = 110, 32%) reported to have previously participated in MDA campaigns. Two third stated their intention to participate (*n* = 232, 66%) or to advise their friends to participate (*n* = 202, 57%) in future MDAs at school. The intention to participate in test and treat campaigns was higher (*n* = 290, 82%). Interestingly the responses to the three questions were inconsistent in over a third of the participants. In order to reduce potential bias (influence of former questions, social desirability bias), only children who gave positive answers (*n* = 138, 40%) in all the three questions were considered as having a high intention to participate in treatment campaigns.

**Fig. 5:**
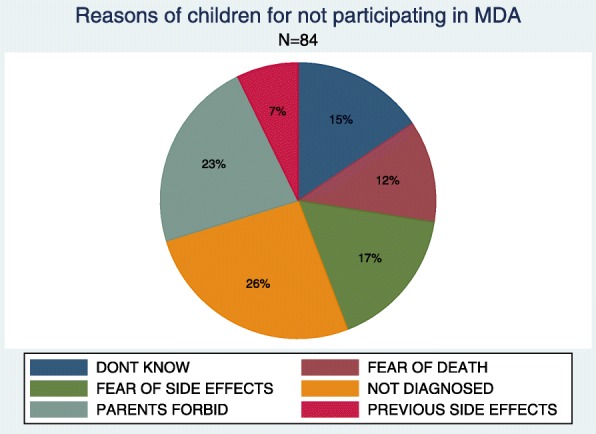
Reasons given by children for not participating in the mass drug administration campaign

Children who indicated no intention to participate in future MDA campaigns mentioned the following reasons: the fear of side effects of the drug; fear of death due to treatment; previous experience of side effects during the previous MDA campaigns (mainly vomiting and stomach pains); and their parents not allowing them to participate due to the parents’ fear of side effects (Fig. 5). Furthermore, children mentioned that, they will not participate in the future MDA if drugs will be offered without prior diagnosis (*n* = 25, 7%).

### Factors associated with relevant knowledge

At bivariate analysis, the following factors were identified to be associated with relevant knowledge about schistosomiasis: i) being aged 14–17 years (OR = 8.4, 95%CI: 2.9–24.5, *P* < 0.001); ii) attending grade 6–7 (OR = 7.6, 95%CI: 3.4–16.8, *P* < 0.001); iii) having heard of schistosomiasis at the school environment (OR = 9.0, 95%CI: 4.5–17.7, *P* < 0.001) or at dispensaries (OR = 6.6, 95%CI: 1.6–26.0, *P* = 0.002).

In addition, children that reported a prior schistosomiasis diagnosis had significantly higher knowledge about the disease (OR = 2.3, 95%CI: 1.2–4.5, *P* = 0.012). Notably, the participation in previous MDA campaigns was not associated with relevant knowledge about schistosomiasis and its preventive measures (OR = 0.8, 95%CI: 0.4–1.5, *P* = 0.5), (Table 2).

**Table 2:** Factors associated with relevant schistosomiasis knowledge among primary school children at Ijinga, northwestern Tanzania

Variables	UnadjustedOR	95%CI	*P*-value	AdjustedOR	95%CI	*P*-value
Sex						
Male	1			1		
Female	1.14	0.65–1.98	0.64	1.04	0.52–2.07	0.91
Age groups (in years)						
5–9	1			1		
10–13	2.23	0.81–6.13	0.11	1.19	0.35–4.10	0.78
14–17	8.35	2.85–24.45	**<0.001**	3.09	0.73–12.98	0.12
Classes						
1–3	1			1		
4–5	2.25	1.02–4.95	0.039**	2.11	0.79–5.66	0.13
6–7	7.55	3.40–16.77	<0.001**	3.94	1.32–11.78	**0.01**
Information Source School						
No	1			1		
Yes	8.95	4.54–17.66	<0.001**	9.94	5.01–19.73	<0.001**
Information Source Hospital						
No	1			1		
Yes	2.56	0.74–8.85	0.12	1.79	0.38–8.32	0.46
Information Source Dispensary						
No	1			1		
Yes	6.61	1.69–25.95	0.002**	3.38	0.69–16.55	0.13
Previous Diagnosis of Schistosomiasis						
No	1					
Yes	2.32	1.19–4.53	0.012**	2.43	1.06–5.55	0.035**
Previous participation in MDA campaign						
No	1					
Yes	0.79	0.42–1.48	0.46	–	–	–

**Significant values are presented in bold

When applying multivariate logistic regression analysis, the factors that remained independently associated with relevant schistosomiasis knowledge were being enrolled in grade 6–7 (aOR = 3.9, 95%CI: 1.3–11.8, *P* = 0.014), school as a source of information (aOR = 9.9, 95%CI: 5.0–19.7, *P* < 0.001), and previous diagnosis of schistosomiasis (aOR = 2.4, 95%CI: 1.1–5.6, *P* = 0.035) Table 2.

### Factors associated with high intention to participate in treatment campaigns

At bivariate analysis, factors associated with the intention to participate in treatment campaigns were being in grade 6–7 (OR = 2.4, 95%CI: 1.4–4.3, *P* = 0.002), aged 14–17 years (OR = 2.1, 95%CI: 1.1–4.0, *P* = 0.016) (Table 3), as well as schools as the source of information (OR = 1.9, 95%CI: 1.2–3.0, *P* < 0.01). Consistent with these findings, children who had relevant knowledge on schistosomiasis (OR = 3.3, 95%CI: 1.8–6.0, *P* < 0.001) and children that had history of participating in MDA (OR = 1.6, 95%CI: 1.0–2.6, *P* = 0.04) indicated higher willingness to participate in treatment campaigns. Other identified factors linked to the intention of participating in treatment campaigns through bivariate analysis were previous diagnosis with schistosomiasis (OR = 3.3, 95%CI: 1.8–6.2, *P* < 0.001), perceiving the disease to be dangerous (OR = 4.1, 95%CI: 2.4–7.1, *P* < 0.001) or deadly (OR = 2.8, 95%CI: 1.8–4.5, *P* < 0.001), the perceived effectiveness of PZQ to cure the disease (OR = 6.3, 95%CI: 3.3–11.7, *P* < 0.001), the perception that the lake is a source for the disease (OR = 3.5, 95%CI: 2.2–5.7, *P* < 0.001), and the own perceived vulnerability of contracting schistosomiasis. Compared to children, who did not perceive themselves as vulnerable towards acquiring schistosomiasis, children who judged themselves at low or high risk of infection had 4.1 (95%CI:2.3–7.4, *P* < 0.001), or 8.5 higher odds (95%CI:3.4–21.2, *P* < 0.001) of intending to participate in treatment campaigns (Table 3).

**Table 3:** Factors associated with high intention of participation in treatment campaigns

Variables	UnadjustedOR	95%CI	*P*-value	AdjustedOR	95%CI	*P*-value
Sex						
Male	1			1		
Female	1.01	0.66–1.55	0.97	1.02	0.61–1.72	0.9
Age groups (in years)						
5–9	1			1		
10–13	1.19	0.69–2.08	0.53	0.99	0.50–1.98	0.98
14–17	2.13	1.13–4.00	**0.02**	1.03	0.46–2.28	0.94
Classes						
1–3	1			1		
4–5	1.06	0.63–1.76	0.82	0.77	0.37–1.60	0.48
6–7	2.42	1.38–4.26	**0.002**	1.06	0.41–2.74	0.89
Relevant knowledge						
No	1			1		
Yes	3.28	1.79–5.99	**<0.001**	1.56	0.68–3.61	0.29
Information Source School						
No	1			1		
Yes	1.88	1.17–3.02	**0.008**	0 .90	0.48–1.70	0.75
Information Source Dispensary						
No	1			1		
Yes	3.14	0.77–12.86	0.09	1.56	0.27–8.96	0.62
Information Source Family						
No	1			1		
Yes	1.73	0.97–3.07	0.05	1.09	0.51–2.33	0.82
Previous participation in MDA campaign						
No	1			1		
Yes	1.62	1.0–2.58	**0.042**	1.00	0.55–1.81	0.99
Previous diagnosis of Schistosomiasis						
No	1			1		
Yes	3.34	1.81–6.16	**<0.001**	2.23	1.05–4.72	**0.04**
Perceived own Vulnerability of getting infected						
None	1			1		
Low	4.09	2.27–7.37	**<0.001**	2.74	1.46–5.14	**0.002**
High	8.54	3.44–21.24	**<0.001**	5.10	2.06–12.60	**<0.001**
Lake considered leading to illnesses						
No	1			1		
Yes	3.52	2.19–5.65	**<0.001**	1.79	1.02–3.14	**0.042**
Schistosomiasis considered dangerous						
No	1			1		
Yes	4.11	2.39–7.07	**<0.001**	2.47	1.28–4.76	**0.007**
Schistosomiasis believed to cause death						
No	1			1		
Yes	2.84	1.78–4.53	**<0.001**	0.77	0.38–1.60	0.48
Medicaments believed to cure the disease						
No	1			1		
Yes	6.25	3.33–11.7	**<0.001**	2.86	1.43–5.69	**0.003**
Medicaments believed to have side effects						
No	1			1		
Yes	1.46	0.89–2.39	0.13	1.09	0.58–2.04	0.79

Significant values are presented in bold

At multivariate logistic regression analysis, there was evidence for an association of high intention to participate in treatment campaigns with: i) previous diagnosis of schistosomiasis; ii) perceived severity of the disease; iii) perceived effectiveness of treatment to cure the disease; iv) perceived general risk of disease transmission by lake water; and v) perceived own vulnerability of getting infected. Children that had been diagnosed with schistosomiasis in the past had 2.2 higher odds (95%CI: 1.1–4.7, *p* = 0.036) of intending to participate in treatment campaigns. The general risk perceived of lake water leading to transmission of disease was significantly associated with the outcome (aOR = 1.8, 95%CI: 1.0–3.1, *P* = 0.042). Children that considered themselves to be vulnerable to schistosomiasis infection because of their lake water contact were 3 (perception of low risk, aOR = 2.7, 95%CI: 1.5–5.1, *P* = 0.002) and 5 times (perception of high risk, aOR = 5.1, 95%CI: 2.1–12.6, *P* < 0.001) more likely to intend participating in treatment campaigns. Perceiving that schistosomiasis was a dangerous disease increased the odds by 2.5 (95%CI 1.3–4.8, *p* = 0.007) and the perception of the treatment being effective in curing the disease increased the odds of planning to participate in treatment campaigns by 2.9 (95%CI 1.4–5.7, *p* = 0.003).

## Discussion

The findings from the presented study revealed that only a small proportion of the school children at Ijinga island had relevant schistosomiasis related knowledge. Most of them expressed no knowledge of *S. mansoni* related manifestations and complications. Factors associated with relevant schistosomiasis knowledge were being in high grades (grade 6–7), school as a source of information about intestinal schistosomiasis and being previous diagnosed infected with schistosomiasis. Less than 50% of the school children demonstrated high intention of participating in treatment campaigns. Factors associated with intention to participate in MDA were being diagnosed with schistosomiasis in the past, perceiving lake water as the source of transmission, perceiving being at low or high risk of being vulnerable to schistosomiasis, perceiving that schistosomiasis was a dangerous disease and perceiving that anti-schistosomiasis were effective in curing the disease.

### Knowledge, perceptions, attitudes and practices

The findings from the present study demonstrated that less than half of the school children reported to have ever heard of schistosomiasis. This was surprisingly low, considering the high prevalence of the disease in the region and that the Island has been the target for control activities for several years. In a previous study among school children in north-western Tanzania, 87.5% of the school children reported to have ever heard of schistosomiasis [29]. In a community based study in the same area, Mwanga et al [27] reported a low level of knowledge about the disease. In many of the studies on the same topic, the general awareness of schistosomiasis is mainly limited to being familiar with the name of the disease and this awareness is always very high [30]. The findings of this study are in line with a recent systematic review paper [30], which has reported a gap on schistosomiasis related knowledge in many of the endemic communities of highly affected countries in sub-Saharan Africa [31].

In our study, school was frequently mentioned as the main source of information about schistosomiasis whereas family or community were only mentioned by a small proportion as a source of information, indicating that schistosomiasis is not a common topic to be discussed on the island. In a previous study conducted in the same region, the main sources of information about schistosomiasis among school children was the school [29]. The school as the main source of information was also reported in Brazil [32]. The observation that communities and the school environment are frequently reported as source of information about schistosomiasis, indicates that these sources of information could act as a good intermediaries to deliver the correct knowledge about schistosomiasis to school children [31]. This is demonstrated by the result of the present study which showed that school children in grade 6–7 who are taught about schistosomiasis in their curriculum demonstrated highest knowledge of schistosomiasis. This observation suggests that inclusion of schistosomiasis in the curriculum of all grades could be an effective strategy to improve schistosomiasis related knowledge among school children. In schools, teachers can be trained about the disease and they should deliver the same knowledge to school children.

On the other hand, specific knowledge about schistosomiasis was very scarce. Less than half of the children related the disease transmission to the lake water contact and only 14% of children could name a correct symptom of the disease. Similarly, in a previous study among school children in schistosomiasis endemic areas, only 10% of the school children associated transmission of schistosomiasis with lake water contact [29]. Misconceptions about transmission of the disease via food and drinking water were also observed in the current study population. A systematic review of Sacolo et al [30] has indicated a lot of misconceptions regarding the transmission pathways of schistosomiasis – revealing that they were often mistaken with the ones related to soil-transmitted helminths. In our study, children also mentioned that their families were boiling or filtering their water to protect themselves from diseases. In relation to schistosomiasis, this misconception can lead to a false sense of security. A lack of perceived vulnerability was also reflected in the low number of children considering themselves at high risk of contracting the disease, despite their frequent or even daily water contact. Other similar misconceptions have been described in previous studies from schistosomiasis endemic communities [27, 30, 33].

Most respondents (86%) were not able to name any correct clinical symptom of the disease.

In Mozambique [34] and Kenya [33], despite the fact that the majority of the respondent reported to have heard about schistosomiasis disease, the knowledge of how the disease is acquired, transmitted, related symptoms and signs was poor. Scarce knowledge on clinical symptoms and signs can lead to a lack of perceived severity of the disease and has been described to decrease participation in repeated MDA and to delayed treatment seeking [35]. This is particularly a concern if the risk of re-infection remains high and treatment campaigns are not expanded to adults. Although most of the children considered schistosomiasis as a dangerous disease in line with previous studies in Tanzania [27, 29] - the severity of schistosomiasis is often not well understood [30, 31], especially due to its slow onset. The low level of awareness of the chronicity of schistosomiasis as a relevant problem has been noted in previous studies [27, 30, 31]. Studies have revealed that schistosomiasis is often considered as a childhood disease that will heal on its own [27, 30]. Immediacy of the onset of symptoms, visibility of symptoms and rate of onset (gradual vs sudden) have been described as relevant dimensions related to the concept of perceived severity [36] and are likely to play a significant role in the secondary prevention of schistosomiasis and the intention to participate in treatment campaigns.

### Factors associated with relevant schistosomiasis knowledge

Our findings on the factors associated with relevant schistosomiasis knowledge indicate that there is a need to revisit the science curriculum to allow topics related to different diseases to be taught at early ages to allow children to recognize these diseases and their preventive measures. If children grow up with this knowledge with repeated emphasize on preventive measures, this might translate to higher participation in control programs such as MDA against various neglected tropical diseases. This might ultimately lead to reduced transmission of these diseases. Studies in tropical areas have noted that schistosomiasis and its related morbidities such as hepatosplenic disease starts at very young age [8, 9, 37]. Thus, it remains important for this education to be introduced to school children from lower classes, giving this education during adolescence age might be too late. Moreover, general awareness and theoretical knowledge on transmission alone are insufficient to induce behavior change if they do not translate into a perception of own susceptibility. Developing a perception of the own vulnerability is a cognitive process taking several phases, from hearing about the existence of the disease threat until personalizing it and estimating one’s own susceptibility [36].

On the other hand, in our present study, reported previous participation in MDA campaigns was not associated with relevant knowledge. According to Lothe et al., [38], it is important that the benefits provided by PZQ are well understood in order to outweigh the fear of side effects caused by treatment. Thus, we endorse that the risk of re-infection and the need for repetition of MDAs should be highlighted during adequate sensitization campaigns beforehand. Otherwise, children might either not feel vulnerable anymore because they consider themselves as being cured or they doubt the effectiveness of treatment [36]. Not addressing these topics and the late complications of schistosomiasis before the start of MDA campaigns can be considered a missed opportunity and could decrease future participation.

### Factors associated with intention to participate in treatment campaigns

Almost all children stated that they would participate in test and treat campaigns. However, children did not seem to have a clear opinion on the usefulness of MDA campaigns without prior testing. This was especially reflected by giving inconsistent answers about their own intention to participation and their advices to friends. These findings are consistent with former projects on Kome Island and in other communities [39]. Even in the presence of high theoretical awareness about schistosomiasis, preceding test and treat campaigns are effective in demonstrating affected communities their own vulnerability and help to engage them in further MDA and other preventive activities [39].

In the multivariate analysis, the only factors associated with high intention of participation in treatment campaigns were questions related to PMT elements, such as perceived vulnerability, severity and treatment efficacy [25]. Protection Motivation Theory elements could be a useful extension of the current KAP models in the African settings endemic for schistosomiasis and should be explored more systematically. Xiao et al., [23, 24] stated that the KAP model might not be sufficient to cover all relevant dimensions necessary for behavior change. In line with this hypothesis, our study showed that relevant knowledge was not directly associated with a higher intention to participate in preventive action. Knowledge might however have an indirect impact through other variables, such as perceived vulnerability and severity as well as the perception of one’s own coping possibilities [25, 27]. Understanding the effectiveness of the intervention and one’s own ability to adopt and maintain preventive behavior is regarded as very important within the PMT framework [25]. Increasing the perception of a personal threat by the disease without one’s ability to cope with it might lead to maladaptive behaviors, such as denial, fatalism and hopelessness [36]. Even though these elements have not been explored in depth during our study, there were prominent indications that they could be relevant, including the open answered questions:

Most children believed in treatment efficacy and surprisingly, only one quarter believed in side effects of Praziquantel. However, a significant number of children stated that their parents would forbid their participation (*n* = 19, 23% of those who would not participate in MDA). A common reason mentioned was the parent’s fear of side effects and death caused by PZQ. This suggests that the children’s willingness to take medicine could be undermined, if the parents are not adequately included in educational activities. Misconceptions about MDA among teachers and parents have been identified as important barriers for MDA uptake in Uganda [18, 20]. In Uganda, the low uptake of PZQ was noted to be the result of a complex interplay between individuals, as well as interpersonal, institutional, community and public policy factors [18]. The individual and interpersonal factors identified were inadequate information about schistosomiasis prevention, beliefs and attitudes in the community about treatment of schistosomiasis and shared concerns among children and teachers about the side-effects of the PZQ, especially when the drug is taken on empty stomach [20]. This indicates that involvement of only parents and teachers may not improve the uptake of PZQ treatment, but the combined involvement of multiple stakeholders is necessary for successful participation of school children in MDA.

An integrated approach, including community participation as well as the provision of wells, water pipes and sanitation infrastructure could increase the motivation for preventive action. Recent examples from China demonstrated that combining several control activities can achieve great results [12].

### Study limitations

The cross-sectional nature of the study design, it was not possible to test for any temporal associations (i.e. whether learning about schistosomiasis in schools led to an improved knowledge of schistosomiasis or intention to participate in MDA). In addition, we only tested some questions related to PMT in an exploratory way and there were examples of no structured questions for the concept of self-efficacy. Thus, the results of this study do not allow any conclusion on the usefulness of PMT as a predictive model for schistosomiasis related behavior in the Tanzanian context. In addition, our findings are only based on one primary school located on the island and this may limit the generalizability of our findings to another African settings endemic for schistosomiasis.

## Conclusions

A proportion of children in higher grades (6–7) had relevant level of theoretical knowledge about schistosomiasis. A small proportion of the children demonstrated high intention to participate in MDA and factors associated with high intention to participate in MDA were previous diagnosis, perceived general risk of disease transmission by lake water, perceived own vulnerability of getting infected, perceived danger of the disease, perceived effectiveness danger of the disease and perceived effectiveness of medicaments to cure the disease.

Schistosomiasis related public health education should be part of the MDA program to increase participation of the children in treatment and impact the knowledge to children in all age groups. In addition, education during school and campaigns should not be limited to talk about general characteristics of the disease, but should also highlight PMT elements, such as the constant vulnerability of each person living in high endemic settings. Lastly, based on our findings, we recommend that studies aiming at assessing KAP should include PMT elements in a systematic manner.

## Supplementary information

**Additional file 1.** Ijinga Schistosomiasis control project: School aged Children Questionnaire.

## Data Availability

The datasets collected and/or analyzed during the current study are available from the corresponding author upon reasonable request.

## Abbreviations

KAP: Knowledge Attitudes and Practices
MDA: Mass Drug Administration
PMT: Protection Motivation Theory
PMT: Protection Motivation Theory
PZQ: Praziquantel
SSA: South Saharan Africa
WHO: World Health Organization
