# Supplementary material for: Factors associated with relevant knowledge of intestinal schistosomiasis and intention to participate in treatment campaigns: a cross sectional survey among school children at Ijinga Island on Lake Victoria, North-Western Tanzania
Source: BMC Public Health. 2019 Dec 30;19:1762. doi: 10.1186/s12889-019-8091-4 (PMC6937638; doi:10.1186/s12889-019-8091-4)
Supplement: Supplementary file 1 — Additional file 1. Ijinga Schistosomiasis control project: School aged Children Questionnaire. [file 12889_2019_8091_MOESM1_ESM.docx]

## Ijinga Schistosomiasis control project: School aged Children Questionnaire

1. Participant information

| N | Participant information | Response |
| --- | --- | --- |
| 1 | Name of data collector |  |
| 2 | District |  |
| 3 | Name of village |  |
| 4 | Name of sub village |  |
| 5 | Name of School |  |
| 6 | Date of participation |  |
| 7 | Name of participant |  |
| 8 | Participant ID |  |

1. Individual Information of participants

| **Sex**  0=Male  1= Female | **Age** | **Current class at school** | **How long have you been living in this village**  0=I was born here  1=I have migrated here | **How many years have you been living here?** | **How many times do you spend more than 2 weeks away from the island?** | **Distance from home to lake** |
| --- | --- | --- | --- | --- | --- | --- |

**Knowledge about Schistosomiasis**

|  | Question | Code | Remarks |
| --- | --- | --- | --- |
| 1 | Have you ever heard of Schistosomiasis? | 0=Yes  1=No |  |
| 2 | What is the common name of Schistosomiasis? |  |  |
| 3 | If yes, where did you hear about Schistosomiasis? | 1=At school  2= Radio/TV  3= Hospital  4= Dispensary  5= Family members (please specify)  6= Others (please specify) |  |
| 4 | How many types of Schistosomiasis do you know? (please mention them) |  |  |
| 5 | How is Schistosomiasis transmitted? | 1= Eating contaminated food 2=Walking in the lake/river  3=Bathing in the lake/river  4=In paddy fields  5=By sexual contact  6=Through Witchcraft  7=Others (please specify)  88=Don’t know |  |
| 6 | Which are the potential areas for transmission of Schistosomiasis? | 1= Lakes  2=Rivers  3=Rice fields  4=Water collection areas  5=Toilets  6= Others (please specify)  88=Don´t know |  |
| 7 | How is Schistosomiasis transmitted from one to another | 1= By witchcraft  2=Urinating in lake/river  3=Defecating in lake/river  4=Contaminating food  5=Sexually  6=Others (please specify)  88=Don’t know |  |
| 8 | Do you know the symptoms of Schistosomiasis? | 0=Yes  1=No |  |
| 9 | If yes, what are the symptoms? | 1=Diarrhea/ bloody diarrhea  2=Blood in urine  3=Swelling of belly  4=Swelling of legs  5=Stomach ache  6=Infertility  7=Vomiting of blood  8=Others (please specify)  88=Don’t know |  |
| 10 | Did you ever have any of the symptoms mentioned above? (Please specify) |  |  |
| 11 | Were you ever diagnosed with Schistosomiasis? | 0=Yes  1=No  88=Don´t know |  |
| 12 | If yes, who did the diagnosis? |  |  |
| 13 | If yes, were you treated? |  |  |

**Attitudes towards Schistosomiasis**

|  | Question | Code | Remarks |
| --- | --- | --- | --- |
| 14 | Do you think that Schistosomiasis is a dangerous disease? | 0=Yes  1=No  88= Don´t know |  |
| 15 | Do you think that Schistosomiasis can cause death? | 0=Yes  1=No  88= Don`t know |  |
| 16 | What is your risk of acquiring schistosomiasis? | 1=High  2= Low  3=None at all |  |
| 17 | Does modern medicine cure/prevent schistosomiasis? | 1=Yes  2=No  88=Don`t know |  |
| 18 | Can traditional medicine cure/prevent Schistosomiasis? | 1=Yes  2=No  88=Don`t know |  |
| 19 | Can drugs for bilharzia cause bad effects on your body? | 1=Yes  2=No  88=Don`t know |  |
| 20 | Can drugs for bilharzia cause death? | 1=Yes  2=No  88=Don`t know |  |
| 21 | Are you likely to advise your friends to take treatment for bilharzia? | 1=Yes  2= No  88=Don`t know |  |

**Practices concerning the disease**

|  | Question | Code | Remarks |
| --- | --- | --- | --- |
| 22 | Do you go to the lake? | 0=Yes  1=No |  |
| 23 | How often do you go to the lake? | 1= Never  2=2-3 times a month  3=Once a week  4=2-4 times a week  5= Once a day  6= More than once a day |  |
| 24 | What is your reason for going to the lake? | 1=Fetching water  2= Fishing  3= Washing clothes/Utensils  4= Bath/Swim/Play  5= Transport  6= Others (please specify) |  |
| 25 | What is your main source of water? | 1= Lake  2=Shallow well  3=Borehole  4=Tap  5=Others (please specify) |  |
| 26 | Is your family treating the water before drinking? | 0=Yes  1=No  88= Don´t know |  |
| 27 | If yes, how do you treat it? |  |  |
| 28 | Do you think going to the lake more frequently can lead to transmission of disease? | 0=Yes  1=No  88=Don`t know |  |
| 29 | If yes, which type of diseases can be transmitted through lake water contact? |  |  |
| 30 | How do you prevent yourself from getting Schistosomiasis? | 1= take a pill  2=Eat healthy food  3=Avoid swimming/bathing in lake  4=Wear rubber boots while in contact with water  5=Others (please specify)  88=Don`t know |  |
| 31 | If free diagnosis, examination and treatment for Schistosomiasis was offered on the island, would you participate? | 0=Yes  1=No  88=Don`t know |  |
| 32 | If no, why would you not participate? |  |  |
| 33 | Have you ever heard of the provision of mass drug administration against Schistosomiasis? | 0=Yes  1=No  88=Don`t know |  |
| 34 | Have you ever participated in mass drug administration against Schistosomiasis? | 0=Yes  1=No  88=Don`t know |  |
| 35 | If mass drug administration against Schistosomiasis is offered on the island without diagnosis, would you like to participate? | 0=Yes  1=No  88=Don`t know |  |
| 36 | If no, why would you not participate? |  |  |
| 37 | What control measures against Schistosomiasis do you know? |  |  |
| 38 | Do you have a toilet at your house? | 0=Yes  1=No |  |
| 39 | If yes, does everyone at your house use the toilet? | 0=Yes  1=No  88=Don`t know |  |
| 40 | If no, where do other household members defecate/urinate? |  |  |
| 41 | Do you think that the use of toilet can prevent the transmission of Schistosomiasis? | 0=Yes  1=No  88=Don`t know |  |
